# Editorial: Context-Dependent Regulation of Neurogenesis: Common Themes and Unique Features of the Neurogenic Process in Different Model Systems

**DOI:** 10.3389/fcell.2021.678475

**Published:** 2021-04-13

**Authors:** Giuseppe Lupo, Michael Piper, Flavio R. Zolessi

**Affiliations:** ^1^Department of Biology and Biotechnology “C. Darwin”, Sapienza University of Rome, Rome, Italy; ^2^School of Biomedical Sciences, The University of Queensland, Brisbane, QLD, Australia; ^3^Sección Biología Celular, Facultad de Ciencias, Universidad de la República, Montevideo, Uruguay; ^4^Institut Pasteur de Montevideo, Montevideo, Uruguay

**Keywords:** neurogenesis, neurogenic niche, neural stem/progenitor cells, neuronal differentiation, *in vitro* and *in vivo* models, model organisms

During neural development, neural stem/progenitor cells (NSPCs) proliferate to self-renew and generate progeny that undergo neuronal differentiation, a process known as neurogenesis. In many organisms, this process is temporally restricted and mostly limited to embryonic and early post-natal development, although adult neurogenesis also takes place in a region-specific and species-specific manner. In the developing nervous system, NSPCs produce region-specific amounts and types of neurons, following distinct temporal schedules, according to their positional identity and the extracellular environment to which they are exposed; these differences are key to shape the anatomical and functional properties of different neural structures, and to ensure proper wiring of complex neuronal networks. Moreover, in adult life, differences in positional information, developmental history, and extrinsic cues across the nervous system determine whether or not specific neural structures are capable of continued neurogenesis, as well as the quantity and the fate of adult born neurons.

Over the years, molecular studies have shown that the core mechanisms driving NSPC self-renewal and neuronal differentiation are remarkably conserved in organisms as different as flies and mice. At the same time, the transcriptional programs expressed in NSPCs, as well as the extracellular signals acting upon them in the neurogenic niche, can change substantially in different organisms, or even in different regions or stages within the same organism. This context-dependent regulation of neurogenesis confers unique properties to each neurogenic niche and its neuronal output. Grasping both the shared and the unique mechanisms underlying NSPC function and neurogenesis in different contexts may crucially improve our understanding of how the exceptional cellular, anatomical, and functional complexity of the human brain is achieved and maintained in the developing and adult organism. It would also be paramount to the development of experimental models that faithfully recapitulate this complexity *in vitro*, and of cell therapies for the treatment of neurodevelopmental and neurodegenerative conditions affecting the nervous system in different regions and ages.

This Research Topic addresses both the fundamental mechanisms of neurogenesis that are conserved in different model systems, and the peculiar traits that distinguish different neurogenic niches from each other, by assembling a remarkable collection of articles written by leading experts in the field. Several of the main model organisms employed in developmental neuroscience are represented in this Research Topic, which includes: (i) key experimental paradigms both in the central and peripheral nervous system (CNS, PNS); (ii) embryonic and adult neurogenesis; (iii) *in vitro* and *in vivo* models. A brief introduction to the articles in this Research Topic is provided below.

Neural development relies on the proliferation and differentiation of NSPCs to be regulated spatially and temporally in a context-dependent manner such that discrete structures can be formed. This is perhaps most clearly exemplified in the mammalian cerebral cortex. This region of the brain has undergone extensive expansion within the mammalian lineage, especially within primates. Understanding how the transcriptional architecture within NSPCs of the anterior brain has changed to facilitate expansion of the neocortex, and how this program has diversified within different mammalian lineages is reviewed by Franchini. This review encompasses research that has asked key questions about neocortical development, including how the neocortex emerged, and how it is different in comparison to brain development in other tetrapods, how the 6-layered structure of the neocortex evolved, and how it is generated, the mechanism and underlying control of cortical gyrification, and the importance of interneurons for cortical function.

Development of the neocortex also requires the coordination of a range of signals, both intrinsic and extrinsic, in NSPCs. For example, Shohayeb et al. describe the role of the spindle microtubule-associated phosphoprotein WDR62 in regulating the proliferation of NPSCs within the developing mouse cerebral cortex. The importance of WDR62 for cortical neurogenesis is underscored by the fact that mice lacking this factor exhibit microcephaly. Conversely, Cao et al. used cultured NSPCs from embryonic rat cerebral cortices to demonstrate that the intravenous anesthetic propofol mediates cell-extrinsic effects on NSPCs, impacting their differentiation. Given the widespread use of propofol, studies such as this are key to understanding how such anesthetics can be safely used. Finally, Xing and Huttner review our understanding of how neurotransmitters within the developing mammalian neocortex serve to regulate NSPC proliferation. This review, as well as a review by Shou et al., also discuss the use of organoids to understand brain development and better define our understanding of neurological diseases. Given that research on the human brain has traditionally been hampered by a paucity of tissue, organoids have emerged as a powerful tool to probe the cellular, molecular, and genetic factors underpinning the development of many regions of the human brain, including the cerebral cortex. Although research has faced issues relating to high variability between organoids, standardization of techniques and protocols is gradually emerging. This has enabled researchers to better understand normal development, as well as to model neurodevelopmental disorders such as primary microcephaly, lissencephaly, and autism in a human context. While challenges remain with organoid technology, such as whether these structures will ever be able to truly reflect the complexity of the human brain, they offer another lens through which the development of the brain can be modeled and tested.

Because of its anatomical accessibility and simplicity, the vertebrate neural retina has long been a preferred CNS area for studying neurogenesis. Three articles of this Research Topic address important aspects of retinal neurogenesis. Retinal NSPCs, like those in other areas of the CNS, are organized in the tight and highly polarized pseudo-stratified neuroepithelium, and they undergo interkinetic nuclear migration as they proliferate. Clark et al. concentrated on the characterization of a particular mechanism influencing the balance between proliferation and neurogenesis of retinal NSPCs in the zebrafish embryo, namely the apical localization of a *Crumbs* family member, Crb2a, depending on the endocytic pathway regulator Rab11a. The generation of a structured and functional retina does not only depend on the balance of proliferation and neurogenesis, since a third player affects the outcome of both processes: apoptosis. This can be physiological, occurring during normal development to adjust neuron number, or pathological, in relation to neurodegeneration. Trying to understand the possible role of DNA damage on retinal degeneration, Gomes et al. inactivated the gene encoding the Rad50 partner protein Rint1 specifically in mouse retinal NSPCs, showing that, instead of causing a proliferation halt by activating the cell cycle checkpoint, this mutation, and the DNA breaks it caused, drove the cells toward apoptosis. Helping to join these two concepts together, the Perspective Article by Oliveira-Valença et al. provides an excellent discussion of an important biomedical issue whose resolution might lie in a better understanding of the basic mechanisms of embryonic neurogenesis: the degeneration of the projection neurons in the retina, retinal ganglion cells, in very prevalent human eye diseases such as glaucoma.

In addition to looking at general mechanisms affecting neurogenesis in whole areas of the nervous system, like the above-described mammalian cortex or the vertebrate retina, there are other levels of analysis. On the one hand, it is important to look at the cellular and subcellular level, where mechanisms tend to be more general across species, organs, and neuronal types. Two reviews in this Research Topic address the important roles of membrane dynamics in neurogenesis. Moore et al. give a brief but essential account on the recently expanding field in cell signaling based on cell protrusions, including filopodia and cytonemes. Interestingly, in addition to secreted paracrine factors, able to diffuse for a variable distance across tissues, and contact between immediate neighboring cells, an increasing number of reports are indicating the presence of a diverse range of cell processes having roles in neurogenesis, such as the ones described here mediating Notch lateral inhibition. Studying the morphological polarization and early differentiation of neurons, Rozes-Salvador et al. analyse the role of intracellular membrane traffic, as modulated by different small GTPases, including Rab11, in neurite outgrowth. On the other hand, a close look into the molecular players regulating the generation and differentiation of one specific cell type is usually very enlightening, as in the case of the review article by Yang et al. on the specification of the enigmatic Kolmer-Agduhr interneurons of the spinal cord of different vertebrate species, which maintain an apical border with a primary cilium toward the ependymal canal.

A different paradigm in vertebrate embryonic neurogenesis is represented by the PNS. In this case, neurons do not arise from the neural tube neuroepithelium, but from cells at the neural plate border (the neural crest; NC) or cranial ectodermal placodes, from which they undergo an epithelial to mesenchymal transition and migrate to eventually populate the peripheral ganglia. Two extensive and complementary reviews discuss the origin, (Mendez-Maldonado et al.), the structural organization (Vermeiren et al.), and the transcription factors involved in neuronal specification (both reviews), of cranial ganglia, while a very interesting research article analyses some of the signals involved in the generation of a set of neurons in the basal chordate *Ciona* (a group lacking NC), which are homologous to dorsal root ganglion neurons (Kim et al.). A special case in the cranial PNS is the olfactory organ, where neurons are themselves exposed to the environment, and are hence expected to be a very sensitive portion of the nervous system to pathogen invasion. In their research report, Palominos and Whitlock explore the relationship between neurogenesis and immune cells in the early development of the olfactory organ in zebrafish.

Following its discovery in mammals, which were thought to generate all their brain neurons during development, adult neurogenesis has become a major focus of neuroscience research, given its involvement in brain plasticity and in neuropathological conditions, and the hope of harnessing it for therapeutic purposes. Since adult NSPCs originate from subpopulations of embryonic NSPCs, and their properties are largely rooted in their developmental history, the need for an integrated view of developmental and adult neurogenesis is increasingly appreciated, as reviewed by Mira and Morante. Focusing on flies and mice, these authors illustrate how the types of cell division and cell interactions used by NSPCs are key to generate neuronal diversity during development and for continued neurogenesis in the adult brain, and how they are influenced by intrinsic transcriptional programmes and extrinsic cues, highlighting the role of niche glial cells.

Although the process of adult neurogenesis has primarily been studied in rodents, other vertebrate models may provide important insights. Labusch et al. review recent progress in the study of adult neurogenesis in zebrafish, discussing how, thanks to adult neurogenic niches easily accessible to live imaging, this organism is allowing to dissect adult NSPC heterogeneity and cell cycle dynamics to a detail difficult to achieve in mammals. Several properties of mammalian adult NSPCs, including their responsiveness to various stimuli, can be recapitulated in zebrafish, and the regenerative capacity of zebrafish neurogenesis may help to understand the lack of regeneration in the mammalian brain. The importance of exploiting the advantages of non-mammalian model systems is also evident in the work of Naef et al., who employed *Xenopus* embryos to show that *Mex3A*, a gene associated with brain aging, is a crucial regulator of NSPC proliferation.

Two reviews focus on classical paradigms of adult neurogenesis in mice: the hippocampal dentate gyrus and the subventricular zone of the lateral ventricles. Bonafina et al. discuss the complexity of the extrinsic cues acting in the hippocampal niche, and the need to further understand how different cues are integrated by NSPCs and/or modulate specific NSPC subpopulations. Ceccarelli et al. examine the effects that various physiological and pathological stimuli exert on adult NPSC proliferation, and their interactions with the genetic programmes controlling the NSPC cell cycle. The authors highlight recent studies showing that appropriate combinations of neurogenic stimuli and genetic modifications can recruit quiescent NSPCs into the cell cycle even in the aged brain, suggesting that the adult NSPC pool may be more resilient to exhaustion than previously believed.

Altogether, this Research Topic provides an updated view of neurogenesis in different regions, stages and organisms, which we hope will be valuable to foster further investigation of this fundamental process and its physio-pathological implications.

## Author Contributions

GL, MP, and FZ equally contributed as Guest Editors of this Research Topic and closely interacted throughout the editorial process, by defining the subjects to be treated and inviting leaders in specific research fields to contribute their work, and by acting as handling editors of all the manuscripts submitted to the Research Topic and writing the Editorial. All authors contributed to the article and approved the submitted version.

## Conflict of Interest

The authors declare that the research was conducted in the absence of any commercial or financial relationships that could be construed as a potential conflict of interest.

